# *Candida albicans* Aortic Fungus Ball in a Behçet’s Disease Patient

**DOI:** 10.34172/aim.34305

**Published:** 2025-06-01

**Authors:** Murat Tasci, Hasan Tahsin Gozdas, Yunus Yilmazsoy, Songul Peltek Ozer, Kemalettin Erdem

**Affiliations:** ^1^Department of Rheumatology, Abant Izzet Baysal University Faculty of Medicine, Bolu, Turkey; ^2^Department of Infectious Diseases and Clinical Microbiology, Abant Izzet Baysal University Faculty of Medicine, Bolu, Turkey; ^3^Department of Radiology, Abant Izzet Baysal University Faculty of Medicine, Bolu, Turkey; ^4^Department of Pathology, Abant Izzet Baysal University Faculty of Medicine, Bolu, Turkey; ^5^Department of Cardiovascular Surgery, Abant Izzet Baysal University Faculty of Medicine, Bolu, Turkey

 A 49-year-old male was admitted with abundant fistulizing purulent discharge from the sternal incision line and left leg pain. He had undergone aortic dissection surgery two years before when the computed tomography angiography (CTA) had shown a type 1 aortic dissection (Figure 1A). He had been receiving antibiotic treatment for the last three months due to suspicion of aortic graft infection, but the response to antibiotic treatment was poor. In the current admission, C-reactive protein was 221 mg/L and erythrocyte sedimentation rate was 70 mm/h. Culture of the sternal discharge and blood yielded no growth. Transthoracic echocardiography did not show any vegetation consistent with infective endocarditis. Current thorax CTA revealed a mass-like lesion in the aortic graft (Figure 1B). Considering this lesion as a thrombus, antiaggregant (cilostazol) and anticoagulant (edoxaban) treatments were begun. Left leg embolectomy was performed due to distal thromboembolism in the left anterior tibial artery. Thereafter, right panuveitis and right ankle arthritis appeared. It was realized that the patient had a persistent moderate elevation in acute phase reactants during the last five years consistent with chronic inflammation. Because of chronic inflammation, panuveitis, arthritis, aortic dissection due to aneurysm and positive pathergy test at 1/6 titer,^1^ Behçet’s disease was diagnosed and intravenous pulse steroid (1000 mg for three days) and intravenous cyclophosphamide 1000 mg were administered. Within two days after this treatment, panuveitis and ankle arthritis disappeared quickly, and a decrease in sternal discharge and a decrease in acute phase reactants were observed. Methylprednisolone treatment was continued at 60 mg/d. Two weeks after admission, his temperatures rose again, the sternal discharge reappeared, and acute phase reactants elevated; so, blood cultures were obtained. In addition, a 10-cm right gluteal hematoma appeared which was drained and sent for culture. *Candida albicans* grew both in the patient’s blood cultures and hematoma fluid which was sensitive to all antifungals in the antibiogram (fluconazole, amphotericin B, caspofungin, micafungin, voriconazole). At this stage, the mass-like lesion in the aorta was thought of as a candida fungus ball rather than a thrombus. Intravenous fluconazole treatment was initiated and the lesion was surgically removed (Figure 1C). Pathological examination of the aortic mass revealed fungal yeast cells and pseudohyphae compatible with *Candida albicans* (Figure 1D). He was transferred to the intensive care unit but expired one week later because of multiple cerebral embolisms.

**Figure 1:**
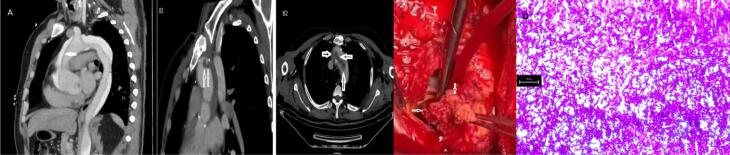
**(A)** Type 1 aortic dissection with an intimal flap (white arrow). **(B1) **Mass-like lesion protruding into the lumen of the aortic arch at the level of surgical anastomosis (white arrows). **(B2) **Extension of the lesion to the aortic arch (white arrows). **(C) **Macroscopic appearance of the lesion taken during surgery. **(D) **Periodic Acid-Schiff-positive fungal yeast cells and pseudohyphae X200

 Aortic vessel wall may weaken and rupture because of chronic inflammation due to various reasons such as Behçet’s disease. *C. albicans* aortic fungus ball is a rare clinical entity which can be encountered in the aorta after aortic graft surgery. Immunosuppression and prolonged antibiotic use may also contribute to development of fungus ball. Treatment consists of systemic antifungal therapy and surgical excision of the lesion. Despite medical and surgical treatment, this serious clinical condition may prove fatal.

*Candida albicans* fungus ball is a life-threatening condition that can develop in the aorta after aortic graft surgery. The main risk factors are weakening of the vascular wall due to aortic graft surgery, immunosuppression and prolonged antibiotic use.^2-5^ Although venous and arterial thromboembolisms are frequently seen in Behçet’s disease, a mass-like lesion in the aorta may not always be a thrombus, and candida fungus ball is a rare possibility. In this paper, we aimed to increase awareness about *C. albicans* aortic fungus ball by reporting this rare clinical entity in a patient with Behçet’s disease.

## References

[1] Davatchi F (2012). Diagnosis/classification criteria for Behcet’s disease. Patholog Res Int.

[2] Tobinaga S, Hirata Y, Saisho H, Wada K, Saku K, Kikusaki S (2016). Successful surgical treatment of a huge Candida albicans aortic fungus ball with pseudoaneurysm. J Heart Valve Dis.

[3] Di Benedetto G, Citro R, Longobardi A, Mastrogiovanni G, Panza A, Iesu S (2013). Giant Candida mycetoma in an ascending aorta tubular graft. J Card Surg.

[4] Kraev AI, Giashuddin S, Omerovic V, Itskovich A, Landis GS (2011). Acute aortic occlusion from a Candida fungus ball. J Vasc Surg.

[5] Kullberg BJ, Arendrup MC (2015). Invasive Candidiasis. N Engl J Med.

